# Consensus Report on *Shigella* Controlled Human Infection Model: Immunological Assays

**DOI:** 10.1093/cid/ciz909

**Published:** 2019-12-09

**Authors:** Robert W Kaminski, Marcela F Pasetti, Ana Older Aguilar, Kristen A Clarkson, Sjoerd Rijpkema, A Louis Bourgeois, Dani Cohen, Ian Feavers, Calman A MacLennan

**Affiliations:** 1 Subunit Enteric Vaccines and Immunology, Department of Enteric Infections, Bacterial Diseases Branch, Walter Reed Army Institute of Research, Silver Spring; 2 Center for Vaccine Development, University of Maryland School of Medicine, Baltimore; 3 Bill & Melinda Gates Foundation, Seattle, Washington; 4 Division of Bacteriology, National Institute for Biological Standards and Control, Potters Bar, United Kingdom; 5 Enteric Vaccine Initiative, PATH, Washington, District of Columbia; 6 School of Public Health, Sackler Faculty of Medicine, Tel Aviv University, Israel; 7 Bill & Melinda Gates Foundation, London, United Kingdom

**Keywords:** *Shigella*, human infection model, immunoassays, vaccine

## Abstract

Moderate to severe diarrhea caused by Shigella is a global health concern due to its substantial contribution to morbidity and mortality in children aged <5 years in low- and middle-income countries. Although antibiotic treatment can be effective, emerging antimicrobial resistance, limited access, and cost affirm the role of vaccines as the most attractive countermeasure. Controlled human infection models (CHIMs) represent a valuable tool for assessing vaccine efficacy and potentially accelerating licensure. Currently, immunological analysis during CHIM studies is customized based on vaccine type, regimen, and administration route. Additionally, differences in type of immunoassays and procedures used limit comparisons across studies. In November 2017, an expert working group reviewed Shigella CHIM studies performed to date and developed consensus guidelines on prioritization of immunoassays, specimens, and collection time points. Immunoassays were ranked into 3 tiers, with antibodies to Shigella lipopolysaccharide (LPS) being the highest priority. To facilitate comparisons across clinical studies, a second workshop was conducted in December 2017, which focused on the pathway toward a recognized enzyme-linked immunosorbent assay (ELISA) to determine serum immunoglobulin G titers against Shigella LPS. The consensus of the meeting was to establish a consortium of international institutions with expertise in Shigella immunology that would work with the National Institute for Biological Standards and Control to establish a harmonized ELISA, produce a reference sera, and identify a reliable source of Shigella LPS for global utilization. Herein we describe efforts toward establishing common procedures to advance Shigella vaccine development, support licensure, and ultimately facilitate vaccine deployment and uptake.

In November and December 2017, workshops were held in Washington, District of Columbia, and London, United Kingdom, by the Bill & Melinda Gates Foundation. The topics discussed focused on immunological assays in support of vaccine efficacy studies and controlled human infection models (CHIMs). Working groups of clinicians and scientists focused on 2 primary objectives during these meetings. The first objective was to reach a consensus on the most appropriate panel of assays to conduct on clinical samples from *Shigella* CHIM studies. These assays will best facilitate assessing vaccine immunogenicity in the context of vaccine efficacy and provide a higher-level understanding of vaccine-induced immune responses, mechanistic principles of protection, and correlates of immunity. The second objective focused on establishing a pathway toward the harmonization and validation of an internationally recognized enzyme-linked immunosorbent assay (ELISA) to determine serum immunoglobulin G (IgG) directed to the O-antigen of several *Shigella* serotypes anticipated for inclusion in future vaccine formulations (*Shigella sonnei* and *Shigella flexneri* 2a, 3a, and 6) using well-characterized antigens, serum controls, and reference standards for data normalization.

Several institutions have been involved with assessment of immune responses in CHIMs and field efficacy studies. The Walter Reed Army Institute of Research (WRAIR), the Center for Vaccine Development (CVD) at the University of Maryland, and Tel Aviv University all have extensive history and experience in the field. Investigators from these 3 institutions conducting *Shigella* immunology were joined by leaders of recent *Shigella* vaccine development efforts and representatives from the National Institute for Biological Standards and Control (NIBSC) and others involved with conducting immunological investigations.

## RECOMMENDATIONS FOR IMMUNOLOGICAL ANALYSES

The workshop held in Washington, D.C., during November 2017 focused on 3 aspects of immunological assays, specifically which assays should be included in *Shigella* clinical studies, the timing of sample collection for these assays, and prioritization of the immunoassays. Clinical study designs are highly customized to the vaccine product being evaluated with generally accepted intervals between prime and booster immunizations specific for administration route and vaccine type (live, subunit). A consensus immunological sampling schedule was developed based on the clinical study design previously used for *Shigella* conjugate vaccines (a 2-dose regimen separated by 28 days) as a framework (Table 1). The interval between last vaccination and oral challenge has not been formalized, but the general agreement in the field is to ensure at least a 28-day interval to allow for the generation of vaccine-induced adaptive immunity and a diminishment of nonspecific, innate immune responses that occur postvaccination.

## ASSAYS TO ASSESS *SHIGELLA* VACCINE IMMUNOGENICITY AND IMMUNE RESPONSES AFTER ORAL CHALLENGE WITH *SHIGELLA* SPECIES

The group discussed immunoassays to evaluate both humoral and cellular immune responses in the context of vaccine immunogenicity and efficacy studies, but with a primary focus on antibody-related assays. Antibodies directed to the O-antigen of *Shigella* lipopolysaccharide (LPS) were thought to be the most essential measurement, as several previous studies have clearly indicated that protective immunity, either vaccine-induced or originating from natural infection, is largely serotype-specific [1–5], and the *Shigella* serotype is determined by the differences in structure and sugars present in the O-antigen of *Shigella* LPS. There were additional discussions regarding the importance of measuring antibodies in both systemic circulation (serum) and in mucosal intestinal compartments, as either direct (fecal samples) or indirect (antibody-secreting cells [ASCs] and antibody in lymphocyte supernatant [ALS]; urine, tears, saliva) measurements. The consensus of the group was that both serum antibodies (IgG, immunoglobulin A [IgA], and immunoglobulin M [IgM], as well as IgG subclasses) and mucosal responses were important.

One portion of the discussion focused on the advantages and disadvantages of ALS and ASCs as indirect measurements of mucosal immunity. ALS assays using antigen-stimulated peripheral blood mononuclear cells (PBMCs) or enriched/purified B cells bearing mucosal homing markers, such as α4β7, would allow for a broader assessment of antibodies directed to multiple *Shigella* serotypes, whereas the number of PBMCs required to measure ASC frequency to the same number of LPS serotypes would be prohibitive. ALS are also easier to perform and less expensive, and lymphocyte supernatants from multiple time points can be batched to reduce assay variability. Therefore, there was a clear preference to conduct ALS rather than ASC assays, especially after bridging studies have been completed for a vaccine program.

Other attributes of the humoral immune response postvaccination or challenge were also discussed. The consensus was that antibody functionality, either in a *Shigella* bactericidal assay (SBA) or opsonophagocytic killing assay, the avidity and affinity of *Shigella* antigen-specific antibodies, and the generation of memory B cells were important parameters necessary to more fully understand immune responses. In addition, the group discussed the value of evaluating the serum antibody response postchallenge by utilizing protein microarrays, which may provide additional insight into antigens recognized after natural infection and potentially allow the identification of novel vaccine targets for improved, next-generation vaccine formulations. In a recent study, a newly developed *Shigella* proteome microarray enabled the identification of novel immune-reacting antigens following oral vaccination and experimental challenge, and a defined type III secretion protein immune profile associated with clinical protection against shigellosis [6].

In relation to the contributions of T cells to a protective immune response, the group consensus was that evaluating T follicular helper (Tfh) cells, in addition to other T-cell phenotypes, by traditional flow cytometry (fluorescence-activated cell sorting) or mass cytometry (CyTOF) would add significant value to the immune assessments. Tfh cells generated shortly after induction of the adaptive immune response have been correlated with memory B-cell generation [7–9], which is a critical immune parameter for effective vaccination and likely relevant for prevention of shigellosis.

In addition to the direct measurements of antibodies in stool, several additional parameters surrounding stool samples were discussed by the group. Stool grading is one hallmark measurement that has been incorporated into *Shigella* CHIM assessments as well as culture-based methods to evaluate shedding of the challenge strain. The group consensus was to also conduct quantitative polymerase chain reaction (qPCR) to quantitate *Shigella* organisms in stool as well as to measure inflammatory markers, such as calprotectin and myeloperoxidase, during the inpatient challenge phase of the CHIM. Elevated levels of fecal inflammatory markers have been associated with physical and cognitive stunting in children [10–13], and the ability of vaccine-induced immunity to blunt the inflammatory response in the gut could be developed as an additional parameter to assess vaccine efficacy, especially in the context of children in low- and middle-income countries. Understanding the effect of *Shigella* vaccination and/or infection on the microbiome was also determined to have value; therefore, collection and processing of fecal samples for eventual microbiome and transcriptome analysis were prioritized.

### SAMPLE COLLECTION TIME POINTS

Collection of biological samples should be adequately timed to ensure effective and accurate immune measurements. Many of them, for example, PBMCs for B-cell measurements, require specific sampling time points postvaccination to ensure capture of mucosally primed cells while they are transiently in circulation and potentially homing to the lymph nodes, bone marrow, or mucosal sites. The kinetics associated with immune responses induced after immunization may be dynamic and a function of route of immunization (parenteral vs mucosal), type of vaccine (conjugate, protein-based subunit, inactivated whole cell, or live attenuated), and whether samples are being collected after a priming or booster immunization. Work conducted in the context of oral immunization or challenge suggests that peak IgA ASC responses occur between 5 and 9 days postintervention [14, 15]. However, practical aspects of outpatient studies, field studies, or studies conducted in small children with significant limitations to sample types and volumes must be considered. For example, the field has largely adapted procedures to collect samples for measurement of mucosal antibodies 7 days postvaccination to align with clinic visits associated with safety assessments. The overall objective therefore was to provide guidance and a framework to be consulted when designing new clinical studies. As such, the working group's efforts focused on developing a clinical time and event schedule utilizing a *Shigella* conjugate vaccine as the example (Table 1).

**Table 1.**
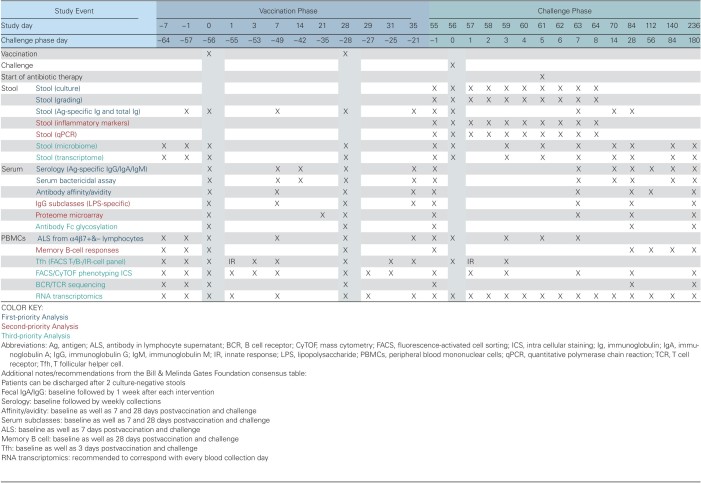
Consensus Time and Event Schedulea

Direct measurements of mucosal immune responses in the intestine are traditionally performed in stool. However, stool samples are susceptible to multiple factors that may confound accurate and reproducible results. For example, transit time through the intestine and volume is individually based, and the temperature and time elapsed from stool production to processing can significantly affect the overall quality of the samples. During the inpatient phase of a CHIM, stool samples can be rapidly transported to the laboratory and processed to prevent degradation, but this is not possible during a typical outpatient vaccination phase. The consistency of specimens can significantly influence results, thus requiring additional specimen treatment (ie, lyophilization) or measurement of total IgA for normalization. In terms of blood collection for PBMC isolation during the CHIM phase, 7 days postintervention is typically utilized to capture peak antibody responses, but also to capture mucosally primed B cells homing to different anatomical compartments. Tfh cells, on the other hand, peak around 3 days postintervention. The inpatient phase of CHIMs facilitates the collection of multiple specimens on a daily basis, which may be essential for measurements of inflammation markers, qPCR analysis, and grading of stool samples. PBMC isolation and serum separation from whole blood may only be necessary at 3 and 7 days postchallenge to adequately evaluate the adaptive immune response induced. Regardless of the availability of immediate funding for immunological analysis, a sampling plan should be developed to effectively collect sufficient samples during the vaccination and CHIM phases of the study for proper processing and archiving, with the intention to secure funding for additional analyses at a later time point as warranted by the clinical study results.

### PRIORITIZATION OF IMMUNOASSAYS

A portion of the workshop focused on building consensus surrounding the prioritization of immunological assays that should be considered when establishing a CHIM study considering that sample types and volumes, subject recruitment, and funding can be limiting factors. The type of specimens obtained from consented subjects largely fell into 3 categories: stool, blood, and others. Stool collections are necessary for multiple immunoassays, but also for bacteriology, microbiome, transcriptome, and inflammatory markers. Blood is typically separated to obtain serum for antibody testing and PBMCs to investigate immunity at a cellular level. In addition to stool samples, various levels of success have also been achieved with saliva, urine, and tear collection, although there is not a general consensus in the field regarding their utility and therefore they were not included in the final recommendations.

After discussion, the working group agreed on a prioritization of immunoassays and placed them into first-, second-, and third-priority analysis tiers (Table 1). Immunoassays within the first-priority tier included the measurement of antibodies in serum and fecal samples by ELISA (or multiplex binding assays) and serum antibody functionality by SBA, as well as the assessment of ALS after separating cells based on homing markers. Assays within the first-priority tier could be considered as primary immunological outcomes in clinical protocol assessments. The second-priority tier includes the evaluation of antigen-specific serum IgG subclasses, protein microarray analysis of serum antibodies, antibody avidity/affinity, and the generation of memory B cells. The third-priority tier, which could be considered exploratory analysis, would be conducted after the first- and second-priority assays were completed and may rely on funding, sample availability, and clinical outcome. Assays within this tier included investigations into the intestinal microbiome, transcriptomics, characterization of the T-cell responses through phenotyping, and evaluation of Tfh cell populations.

### PATHWAY TOWARD AN INTERNATIONALLY RECOGNIZED AND VALIDATED *SHIGELLA* LPS ELISA

The focus of the meeting held in London during December 2017 was ELISA protocols to measure LPS-specific serum IgG. Subject matter experts from the CVD, WRAIR, and Tel Aviv University presented details of their assay procedures and discussed common elements, as well as areas that were unique to each site. The protocols were all generally comparable, but several differences were highlighted, including data analysis, antigen and key reagent sources, and reference or control samples utilized in the assays. The protocols utilized by WRAIR and Tel Aviv University both started from a common protocol and therefore only differed in minor technical aspects, whereas the protocols from WRAIR and Tel Aviv University were less aligned with the protocol from the CVD. One of the major differentiators of the CVD ELISA protocol and those used by WRAIR and Tel Aviv was the utilization of a standard curve to interpolate titers as compared to the WRAIR protocol, which determines endpoint titers by diluting the samples until a negative signal is achieved. Both procedures have their advantages and disadvantages and could be reconciled with minimal effort.

WRAIR outlined their program to produce, qualify, and maintain key ELISA reagents. Purified LPS is isolated from wild-type bacteria using hot phenol extraction methods adapted from Westphal and Jann [16], and quality control performed for each LPS lot includes analysis by silver staining, Western blot for identity using monoclonal antibodies, and nuclear magnetic resonance analysis to determine O-acylation state, as well as DNA and protein content. The purified LPS is placed in a stability program and assessed every year for reactivity using the qualified WRAIR ELISA protocol and a panel of polyclonal and monoclonal antibodies. The LPS manufactured by WRAIR has been used in most phase 1 and phase 2b *Shigella* vaccine studies conducted over the past 10 years, although both Institut Pasteur and Tel Aviv University have also contributed antigen in the context of the STOPENTERICS European Union FP7 program.

WRAIR also described control sera, reference sera, and proficiency panels that have been utilized to deploy the assay across several international laboratories conducting *Shigella* clinical studies, including the International Centre for Diarrhoeal Disease Research (Bangladesh), LimmaTech Biologics Inc, Cincinnati Children's Hospital Medical Center, Johns Hopkins University, the CVD, and the Armed Forces Research Institute of Medical Sciences, Thailand. The consensus from the working group was that LPS antigens were critical reagents necessary for the harmonization of the ELISA and suggested that the product should be made under good laboratory practice or good manufacturing practice conditions, in sufficient quantity to be used globally for a decade or more, and be readily available to all laboratories that will conduct immunoassays on samples collected in support of clinical studies.

The workshop attendees discussed next steps in the process of establishing an internationally recognized ELISA. Presentations by representatives from the NIBSC outlined the mission, capacity, expertise, and experience with establishing and distributing immunoassays and the necessary reference samples, controls, and protocols based on their existing relationship with the World Health Organization (WHO) on numerous projects. The NIBSC has recently conducted a collaborative study involving multiple international laboratories, which confirmed the suitability and assigned unitage to a newly prepared WHO Anti-Vi Reference Serum Standard, and compared the performance of different Vi ELISA formats across multiple sites [17]. The consensus from the workshop was that the NIBSC could similarly create and facilitate a protocol for comparison and harmonization of LPS-specific ELISAs within the major institutions that conduct *Shigella* immunoassays, with a focus on *S. flexneri* 2a and *S. sonnei* to precede *S. flexneri* 3a, 6, and 1b.

A consortium will be created including WRAIR, CVD, Tel Aviv University, Johns Hopkins University, and GSK Vaccines for Global Health, with logistic support and organizational efforts led by the NIBSC. Each consortium member will provide the NIBSC with standard operating procedures (SOPs) of their in-house ELISA and sera with low, medium, and high anti-LPS IgG titers. These materials will be confirmed to be free of blood-borne pathogens, freeze-dried, and distributed in coded format, along with essential reagents and components of the assay, within the consortium. Each consortium laboratory will test the study samples in their in-house assay, in the first instance, and provide data sets to the NIBSC for statistical analysis including intralaboratory and interlaboratory reproducibility, repeatability, and accuracy using appropriate statistical methodology. The objective is to achieve a protocol for a harmonized ELISA and the production of adequate supplies for LPS antigen for plate coating, reference and control serum, and other key reagents for the global deployment of the harmonized assay. The NIBSC will work with consortium members to establish a detailed SOP of the harmonized LPS ELISA, guidelines for data analysis, endpoint titer calculation, and data reporting, and will facilitate central distribution of reagents and tools to further ensure consistency across multiple laboratories conducting *Shigella*-specific ELISAs.

## CONCLUSIONS

The overall objective of the 2 workshops organized by the Bill & Melinda Gates Foundation was to help advance second-generation O-antigen–based *Shigella* vaccines by establishing consensus in the field regarding specimen collection, prioritization of *Shigella*-specific immunoassays, and harmonization of procedures to evaluate serum IgG responses directed to *Shigella* LPS. A *Shigella* vaccine remains a top priority for the global community, both for travelers and for children who live in endemic areas of the world. As *Shigella* vaccine candidates progress through the clinical pipeline, establishing a well-thought-out framework to guide immunological investigations will facilitate the comparison of vaccine-induced immunity and assist in the selection and advancement of effective *Shigella* vaccine candidates. While work progresses toward the establishment of a harmonized ELISA, members of the consortium will work with the NIBSC to ensure global access to the necessary protocols, procedures, reagents, and tools required to generate results that can be used across multiple efforts focused on the common goal of producing an effective *Shigella* vaccine for the world.

## Notes

***Disclaimer.*** The views expressed in this article do not necessarily reflect the official policy or position of the Department of the Army, the Department of Defense, nor the US government. This is a partial US government work. There are no restrictions on its use. Two of the authors (R. W. K. and K. A. C.) are employees of the US government. This work was prepared as part of official duties. Title 17 U.S.C. §105 provides that “[c]opyright protection under this title is not available for any work of the United States government.” Title 17 U.S.C. §101 defines a US government work as a work prepared by a military service member or employee of the US government as part of that person's official duties.

***Supplement sponsorship.*** This supplement is sponsored by the Bill & Melinda Gates Foundation.

***Potential conflicts of interest.*** The authors report no potential conflicts of interest. All authors have submitted the ICMJE Form for Disclosure of Potential Conflicts of Interest. Conflicts that the editors consider relevant to the content of the manuscript have been disclosed.
